# The perceived importance of prognostic aspects considered by physicians during work disability evaluation: a survey

**DOI:** 10.1186/s12911-022-01758-0

**Published:** 2022-01-29

**Authors:** Sylvia P. Snoeck-Krygsman, Frederieke G. Schaafsma, Birgit H. P. M. Donker-Cools, Carel T. J. Hulshof, Lyanne P. Jansen, René J. Kox, Jan L. Hoving

**Affiliations:** 1 Department of Public and Occupational Health, Amsterdam Public Health Research Institute, Amsterdam UMC, Location Academic Medical Centre, Meibergdreef 15, 1105 AZ Amsterdam, The Netherlands; 2 Research Center for Insurance Medicine (KCVG): collaboration between AMC–UMCG–UWV–VUmc, Amsterdam, The Netherlands; 3 Department of Social Medical Affairs (SMZ), The Dutch Social Security Institute: The Institute for Employee Benefits Schemes (UWV), La Guardiaweg 94-114, 1043 DL Amsterdam, The Netherlands; 4 Department of Public and Occupational Health, Amsterdam Public Health Research Institute, Amsterdam UMC, Vrije Universiteit Amsterdam, Van der Boechorststraat 7, 1081 BT Amsterdam, The Netherlands

**Keywords:** Prognosis (MeSH), Work (MeSH), Disability evaluation (MeSH), International classification of functioning, Disability and health (MeSH), Evidence-based medicine (MeSH)

## Abstract

**Background:**

Assessing prognosis is challenging for many physicians in various medical fields. Research shows that physicians who perform disability assessments consider six areas when evaluating a prognosis: disease, treatment, course of the disease, external information, patient-related and physician-related aspects. We administered a questionnaire to evaluate how physicians rate the importance of these six prognosis areas during work disability evaluation and to explore what kind of support they would like during prognosis assessment.

**Methods:**

Seventy-six physicians scored the importance of 23 prognostic aspects distributed over six prognosis areas. Participants scored the importance of each aspect both “in general” and from the perspective of a case vignette of a worker with a severe degenerative disease. The questionnaire also covered needs and suggestions for support during the evaluation of prognoses.

**Results:**

Medical areas that are related to the disease, or the treatment or course of the disease, appeared important (scores of 7.0–9.0), with less differing opinions among participants (IQR 1.0–3.0). Corresponding verbatim remarks supported the importance of disease and treatment as prognostic aspects. In comparison, patient- and physician-related aspects scored somewhat lower, with more variability (range 4.0–8.0, with IQR 2.0–5.0 for patient- and physician-related considerations). Participants indicated a need for a tool or online database that includes prognostic aspects and prognostic evidence.

**Conclusions:**

Despite some variation in scores, the physicians rated all six prognosis areas as important for work disability evaluations. This study provides suggested aids to prognosis assessment, including an online support tool based on evidence-based medicine features.

## Background

Prognosis is the forecast of an outcome, and in a disability evaluation this outcome mainly concerns the functional abilities needed for work [1]. For example, will a 50-year-old nurse with degenerative knee arthritis be able to climb stairs in the future? [2, 3]. The evaluation of the prognosis is an important aspect of disability evaluations [4, 5] and has financial, personal, and legal consequences. Prognosis assessment is internationally considered to be one of four main tasks in disability evaluations [6], but physicians find the assessment of prognosis challenging [7, 8]. Prognostic questions make up 39% of physicians’ information needs during disability evaluation [9].

A Dutch study identified 23 aspects for consideration in the prognosis evaluation during disability assessment [8]. These aspects fell into six areas: the specific disease or disorder, any treatment or potential treatment, the course of the disease, external information from other specialists or scientific evidence, patient-related aspects (e.g., coping), and physician-related considerations (e.g., role, empathy). However, it is not known how these aspects are valued and dealt with in practice. Although this qualitative study provided us with a range of aspects considered during prognosis assessment, it did not provide quantitative information on how important physicians rate these aspects or why some are considered more or less important. More insight into the importance of the aspects considered during prognosis assessment will also guide the development of a prognostic tool in the future.

Therefore, the purpose of this study was to evaluate how physicians rate the importance of six prognosis areas considered during work disability evaluation and to explore what kind of support they would like during prognosis assessment.

## Methods

### Design

In this study, we asked participants in a workshop to complete an anonymous paper questionnaire.

### Participants

The study participants includes mainly social insurance physicians and occupational physicians, who assess a person’s work capacity in the context of a disability benefit claim or with respect to reintegration, respectively. The participants attended an annual congress of the professional association for social insurance medicine in the Netherlands. The programme comprised several lectures and workshops, e.g. on prognosis assessment. This workshop involved a plenary presentation, followed by a case demonstration, discussions and the completion of an anonymous questionnaire. All procedures followed were in accordance with the ethical principles for medical research involving human subjects: before participants attended the workshop, they were informed about the study aim and procedure. Participation was voluntary; participants had the opportunity to discontinue at any time.

### The questionnaire

**Fig. 1:**
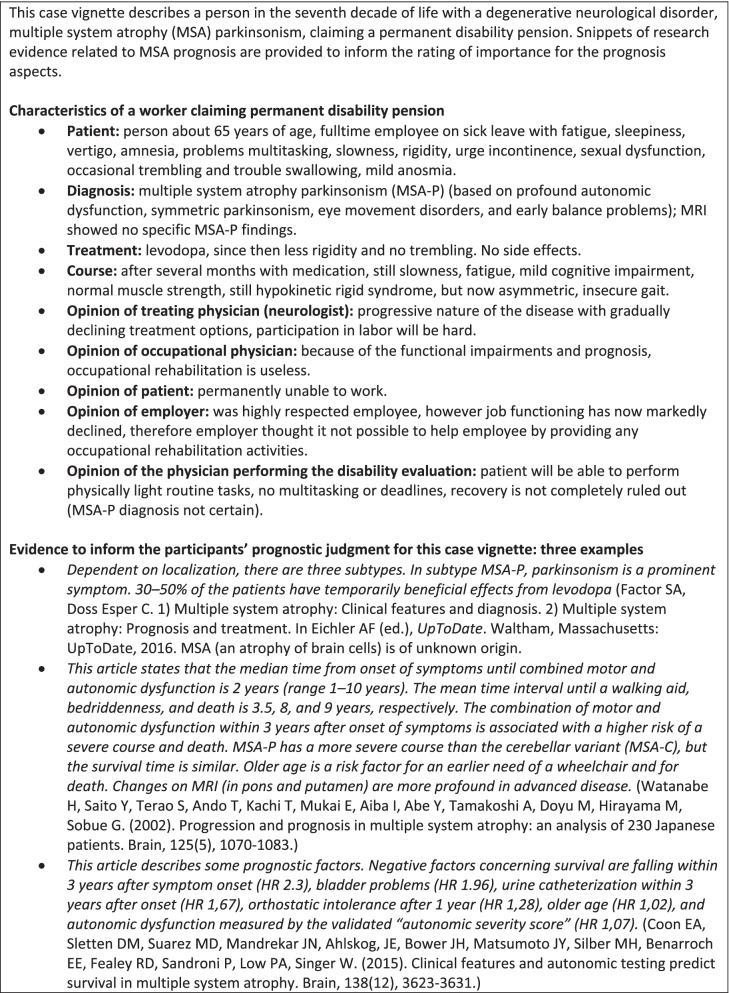
Case vignette and examples of evidence provided to physicians

Two authors (RK, JH) developed the questionnaire specifically for this study, it consisted of three parts. The first presented the 23 prognosis aspects found by Kox et al. [8]. The physicians were asked to rate the importance for the prognosis on a Likert scale ranging from 1 (*not important at all*) to 10 (*of utmost importance*), both for the presented case vignette (see Fig. 1) and “in general,” that is, for any kind of patient. Each aspect was followed by an open question asking participants to motivate their answer, for example: “How important, in your opinion, is the severity of the disease for the prognosis? Please give a rating on a scale of 1 (*not important at all*) to 10 (*of utmost importance*) for this case vignette and in general.” The second part consisted of five questions. Four were about encountered needs and solutions for the assessment of prognosis. The fifth asked whether the physician judged that the assessed limitations in functioning for the case vignette were permanent. The last part of the questionnaire consisted of questions concerning the respondent’s characteristics, for example, age, sex, job area (e.g., social insurance medicine or occupational medicine), years of experience performing disability evaluations, and number of hours worked per week.

### Statistics and analyses

The answers were stored and analyzed in IBM SPSS Statistics for Windows®, version 25.0 (SPSS Inc., Chicago, IL, USA) using descriptive statistics. As most of the dependent variables from both the Likert scale questions and the demographic questions were not normally distributed, we calculated the median and interquartile range (IQR) to describe the distribution of the scores. The open answers were read and entered into SPSS by LJ; two researchers (SK and BD) double-checked these data. For the qualitative analyses, one researcher (SK) explored the answers and grouped them into themes; this process was then checked by three other researchers (JH, FS, BD, and LJ.)

## Results

### Demographics

Of the 78 physicians attending the workshop, 76 completed the questionnaire and handed it in after the workshop. The demographic questions in the last section of the questionnaire were completed by 53–55 of all responders. Most of the participating physicians worked in social insurance medicine (n = 35) or in occupational medicine (n = 15). Three participants worked in both these specialties and two worked in another medical specialty. A slight majority of the physicians were over 55 years old (n = 31, versus n = 23 less than 55 years), most had been in the profession for over 15 years (n = 42, versus n = 12 less than 15 years), and most worked 32 h or more per week (n = 40, versus n = 13 less than 32 h).

### Ratings of prognosis aspects relevant for the assessment of prognosis

When the physicians were asked to rate the importance of the prognosis aspects “in general,” their median scores varied between 6.0 and 8.0 (see Table 1). The median importance scores concerning the same prognosis aspects for the case vignette (person with MSA parkinsonism) were between 4.0 and 9.0. Interquartile ranges (IQRs) varied between 1.0 and 4.8 “in general” and between 1.0 and 5.0 for the case vignette.

**Table 1:** Importance scores of prognosis aspects (n = 76), “in general” and for the case vignette

Area	Prognosis aspects worth considering when evaluating disability prognosis	Importance (1–10) “In general”		Importance (1–10) Case vignette	
		Median	IQR	Median	IQR
1. Disease	Nature	8.0	1.0	8.0	2.0
	Severity	8.0	1.3	9.0	2.0
2. Treatment	Type	8.0	2.0	8.0	2.0
	Effect	8.0	1.0	8.0	2.0
	Alternatives	8.0	2.0	8.0	3.0
3. Course	Course	8.0	1.0	8.5	1.0
	Cause and disease maintaining factors	8.0	1.5	7.0	3.0
	Aspects of revalidation and vocational rehabilitation	8.0	1.5	7.0	3.0
4. Information and evidence	Information from specialist/professional	8.0	2.0	8.0	1.0
	Evidence from literature/guidelines/protocols	8.0	1.3	8.0	2.0
5. Patient-related considerations	Patients own vision concerning recovery	8.0	4.0	6.0	5.0
	Work perspectives	7.0	3.0	7.0	4.0
	Indirect advantage of being ill	6.0	4.5	4.0	4.0
	Significance of work	7.0	3.0	5.0	4.3
	Recovery behavior	7.5	2.0	6.0	4.8
	Coping regarding disease or changed role	8.0	2.0	6.0	4.0
	Psychosocial factors	7.0	2.3	5.0	4.3
6. Physician-related considerations	Perceived role	7.0	3.0	5.0	4.0
	Empathy/compassion for the patient	6.0	4.5	6.0	4.0
	Medical ethics	6.5	4.0	7.0	4.8
	Influence of employer, colleagues, office culture	6.0	4.8	5.0	4.8
	Patient observation and related physician impression	7.0	2.0	7.0	3.0
	Anticipation of outcome	6.0	3.0	6.0	4.5

The number of answers varied per question, ranging from 64 to 76 (= 84–100% of the participants)

For the prognosis aspects scored “in general,” the first four prognostic areas (disease, treatment, course, and information) had median scores of 8.0, with IQRs of between 1.0 and 2.0. The remaining two areas—namely patient- and physician-related considerations—had more variable scores (i.e., medians ranged between 6.0 and 8.0), with IQRs of between 2.0 and 4.8.

In comparison, the case vignette had a somewhat higher importance score for severity of the disease (9.0), while prognosis aspects—such as disease-maintaining factors and vocational rehabilitation—had a somewhat lower score (7.0). From the interquartile ranges (IQRs), it can also be seen that physicians varied more in their assessment of, in particular, the patient- and physician-related considerations. Scores in the first four areas ranged between 7.0 and 9.0 with IQRs ranging from 1.0 to 3.0, whereas scores in the latter two varied between 4.0 and 7.0 with IQRs of between 3.0 and 5.0.

### Open questions on the motivation for the ratings

Illustrative answers are shown in Table 2.

**Table 2:** Motivations accompanying the scores of importance (open questions) [Translated by the authors. Original source consisted of Dutch handwritten texts.]

Area	#	Quotes by participants
1. Disease	#42	For prognosis of future functioning, [the] disease (nature plus^a^ severity) is [the] starting point for [the physician (]OP/IP^b^[)]. […]
	#32	Main point being whether or not the disease is progressive
	#73	In the case vignette, it concerns a progressive disease; that certainly is determining for the prognosis. In general, there are also situations in which the patient’s coping is also an important factor, in addition to nature/severity of the disease
2. Treatment	#6	If there are several treatments available after the treatment that the client^c^ is currently receiving, then [there] may be a chance of improvement [of] work capacity
	#27	[Treatment] [e]ffect; can be [an] indication for influencing progression. [Judgment on treatment] [a]lternatives; necessary for me for *IVA*^d^. No alternatives left equals^e^ game over (end stage)
	#26	In progressive diseases you are postponing [the] final state. In general: are there still valid treatment options?
	#52	Treatment in this case vignette is aimed at treating symptoms. Not curative
3. Course	#4	Course, the past, tells something about the future
	#27	Progressive disease, you want to know where [the patient] is on the downward line. The rest is not that important
4. Information and evidence	#15	*C/* [Conclusion] of the treating neurologist (progressive complaints of illness)
	#23	Evidence preponderates, [it] excludes bias in [the] treating physician
	#19	Evidence [is] not necessary in most cases, because [they are] familiar, routine
	#36	Especially with a substantial claim ([full disability pension/benefit,] IVA \* MERGEFORMATd): info, evidence
5. Patient-related considerations	#23	With a positive attitude [of the patient], the prognosis can turn out to be more favorable than the expectation [of the prognosis for that disease mentioned] in the evidence
	#45	How the patient perceives^f^ [his or her] complaints and^g^ limitations plus \* MERGEFORMAT a advantages of being ill, coping determines the prognosis for recovery
	#51	Given [the] age [of the] client \* MERGEFORMAT c, I would assign [a full disability pension/benefit,] IVA \* MERGEFORMAT d
	#18	Age. To evaluate doesn’t equal^h^ “to take into account.”^i^ Quality of life when client [is] continuing [to] work
	#26	In general, these are rather predictive aspects for success in vocational rehabilitation. Not often part of the considerations [for] prognosis
	#46	[It] [i]s derived from literature that patient’s opinion on duration of incapacity for work is an important prognostic factor. On the other hand, the perception of the patient can be influenced by the messages physicians provide. So, I see [the] perception of [the] patient as important and as influenceable
6. Physician-related considerations	#72	Own impression plusa assessment is also very important on [*sic*] the individual client, in addition to the info practitioner plusa available evidence
	#32	I try to take these factors into account as little as possible
	#50	[There] is [a] good chance that [the physician,] OP/IPb[,] takes “ethical” [*sic*] aspects into account; and certainly not if there is a less serious/threatening condition
	#73	As [a physician performing disability evaluations,] IP^j^[,] you have to deal with a legal framework. The judgment must be based on medical examination. The impression of the client can in my opinion^k^ not always be leading. Especially since there’s a financial interest. [This] [a]lso applies to eg [*sic*] empathy/compassion for the client. […]
	#45	Physician bias plays a role too in assessing the prognosis
	#42	A client should receive the same outcome with every [individual physician,] OP/IPb. Role interpretations OP.IP [*sic*] play no role then. I notice that I take patient’s age into account. Say [the patient is] 25 years old…
	#24	Difficult to assign [a] gradation [to the] importance, because many factors are subconscious [“unconscious” in original]
	#29	Is influence [of] your personal blind spot too
	#27	I base prognosis in [*sic*] a theoretical framework. I try to be as objective as possible, but aware of my own experiences

^a^Plus symbol in original

^b^Occupational physician and/or (social) insurance physician; both assess disabilities for work

^c^Patient in social security setting claiming disability benefits

^d^Permanent disability pension

^e^Equality symbol in original

^f^Brackets in original replaced by parentheses

^g^Ampersand in original

^h^Inequality symbol in original

^i^Quotation marks added for clarity

^j^Insurance physician. Evaluates functional abilities for work for claimants requesting disability benefits

^k^Abbreviated in original

#### Disease

Some physicians stated that the nature and severity of the disease are a starting point for the assessment of the prognosis (e.g., #42). Several physicians made it explicit that the degenerative, progressive nature of the disease is essential for the prognosis (e.g., #32). It was also noted that in the case vignette, the progressive nature of the disease was most important for prognosis, whereas in other cases other prognosis aspects might also be relevant (e.g., #73).

#### Treatment

Considering treatment, many physicians evaluated the effect of the current one and looked for future options that could bring about a change (e.g., #6, #27). It was also pointed out that one should distinguish short-term treatment effects from long-term disease outcomes. In addition, physicians distinguished between relief of symptoms and curation (e.g., #26, #52).

#### Course

Regarding the course of the disease, physicians considered that the rate of progression of the disease and the results of treatment up to the moment of the disability evaluation forecast future developments (e.g., #4). For the case vignette with a progressive medical disease, it was mentioned that the course of the illness (amount of deterioration over time) was of main importance (e.g., #27).

#### Information and evidence

Information from the treating physician is considered important to get an insight into the patient’s unique course and progressive symptoms (e.g., #15). Some physicians stated that they preferred research evidence (e.g., #23). Others pointed out that you may not need scientific literature in all cases (e.g., #19), but you may need more evidence when a patient claims a large number of ongoing disabilities (e.g., #36).

#### Patient-related considerations

Several patient-related factors, such as coping and the way patients perceive their illness, were considered important (e.g., #23, #45). Others spontaneously mentioned age as a patient-related ADP that either guides the decision (#51) or does not (#18). One physician (#26) stated that patient-related factors can be important for vocational rehabilitation, but that they inform the physician conducting a disability evaluation to a lesser degree. Physicians also mentioned that it was important to assess whether patient-related prognosis aspects can be changed (e.g., #46).

#### Physician-related considerations

Regarding the importance of physician-related factors, differing opinions were found, ranging from “very important” (e.g., #72) to “as little as possible” (e.g., #32), and some mentioned them to be case-dependent (e.g., #50, #73). Some physicians said that ethical aspects were considered (e.g., #50) and that the physicians’ own impressions were important (e.g., #72), while others indicated that these should not be a part of the evaluation as they could unintentionally influence their judgment (e.g., #73, #32) or could bias the physician (e.g., #45), and efforts should be made to ensure that a client receives similar outcomes from different assessors (e.g., #42). However, it was also stated that these prognosis aspects are often hard to recognize, given their often subconscious nature (e.g., #24). They are interrelated with a physician’s “blind spot” (#29), but it is important to be aware of those (e.g., #27).

### Specific support that physicians suggest would aid them during the prognostic assessment

Several answers illustrate additional needs, suggestions, and support for physicians during the assessment of prognosis (see Table 3). Principles of evidence-based medicine (EBM) were suggested as an integral part of the support (e.g., #45, #73), which could include evidence-based information on the nature (e.g., #45) or course of a disease (e.g., #73), treatment options, (e.g., #45, #73) and prognostic factors (e.g., #8, #16, #32, #77) that could be supported by figures, facts, and references (e.g., #46). Some physicians mentioned that a potential tool should provide information about relevant prognosis aspects (e.g., #58). The case vignette used might illustrate relevant prognostic information (e.g., #41) as an example. A diagrammatic way of presenting information (such as checklists, programmed instructions, or flow charts) was also suggested (e.g., #17).

**Table 3:** Potential elements of supportive aid for prognosis assessment (open and checkbox questions)

Ingredients: What should a prognosis assessment method provide you with?	#46	A simple instrument that can easily be used in practice
	#73	It must be evidence-based, identify treatment options for the disorder, course of the disease
	#8	I would prefer to have a document in which the pros and cons of the forecast are weighed on the basis of current evidence
	#77	Model to weigh factors
Information in prognosis aid: What information should this prognosis assessment aid contain?	#41	All items mentioned in the case vignette
	#58	Summary of relevant aspects
	#45	Evidence-based information, info on the nature [of the] disease in time plus^a^ therapeutic options
	#46	Figures, facts, and substantiation (references)
	#55	All necessary: (1) [is there complete work disability according to the labor expert]^b^ (2) search path (to be copied) in case of [a] possible [search in] PubMed/Medline or other search (3) info [*sic*] about prognosis of the most common diseases (from relevant literature such as systematic reviews)
	#16	[T]he most important factors you should take into account and what weight they carry for your judgment
	#32	Summary of known research about the disease. Prognostically favorable/unfavorable factors
	#17	[I]nstruction or checklist in a general sense. [W]ith psychological complaints pay attention to a, b, c etc.[,] with neurologic [ones]: pay attention to a, b, c, etc. So disease-related

^a^Plus symbol in original

^b^Substituted for abbreviated jargon in original

#### Type of aid

As a preferred medium, digital aids were most frequently chosen (e.g., “website” n = 38, “app” n = 17). A desk pad format (a leaflet summarizing key remarks) was also considered useful (n = 16).

## Discussion

In this study we evaluated how physicians working in the field of disability evaluation rated the importance of six areas of prognosis, namely disease, treatment, course of the disease, external information, patient-related aspects, and physician-related aspects. Although all six areas were considered important, there was more consensus among physicians concerning the three medical areas (disease, treatment, and course). The scores and verbatim remarks regarding the patient- and physician-related considerations (non-medical areas) reflected a more varied appreciation of importance among physicians.

Our use of a clear and severe medical case vignette may have influenced the physicians’ more limited appreciation for non-medical prognosis aspects during the prognosis assessment. Verbatim remarks were made to the effect that medical considerations sufficed, and the importance of medical prognosis aspects appeared relatively high. In contrast, physicians’ remarks regarding prognosis assessment of cases “in general,” suggested that non-medical prognosis aspects could become more important in cases with a less clear medical background. Physician-related considerations appeared not to be given an explicit role in the assessment. Some physicians actually mentioned this and scores within this area appeared lower. However, it was also mentioned that they do play a relevant, though often implicit or subconscious, role.

As a form of support, physicians mentioned some kind of overview of prognosis aspects and relevant scientific evidence. A digital form of support was preferred.

### Comparison with the literature

#### The importance of the six areas of prognosis: diverging opinions on non-medical prognosis aspects

In disability evaluations, the framework of the International Classification of Functioning, Disability and Health (ICF) [10] has been adopted by and used in several countries [11, 12]. This classification system can also be used to describe work functioning, taking contextual and personal factors into account. However, there is some criticism that the ICF scheme is suggestive of the dominance of a medical perspective rather than a biopsychosocial one. Therefore, suggestions for a revision of the ICF have been made [13, 14]. For prognosis assessment in work disability evaluations, there is another problem with the ICF: The dynamic aspect of functioning and health over time is not addressed, nor are any consequent changes in activities or participation over time. As such, the use of the ICF during social–medical history taking and prognosis assessment is limited [11]. These two issues with the ICF (i.e., suggestive dominance of the medical perspective and absence of a time frame) leave room for diverging opinions [15, 16], especially regarding non-medical aspects, which corresponds to our findings.

#### The importance of the six areas of prognosis: non-medical prognosis aspects

Our study shows that the perceived relevance of patient-related considerations varies in prognosis assessment. In the case of a clear medical condition, they might not all be necessary or might make a smaller contribution to the prognosis evaluation, whereas for medically unexplained physical symptoms (MUPS) or in vocational rehabilitation settings, physicians may attribute more value to them.

In a study on arguments used in disability prognosis [17], *medical clarity* contributed to the type and number of arguments used. In less clear medical cases, more arguments were used, including non-medical aspects such as coping or education. In addition, Ankersmit et al. [17] found that the *expected outcome reception* played a role: when disability claims were substantial or the chances of appeal were considered high, physicians preferred a more comprehensive evaluation of all potentially relevant aspects, including patient-related considerations (a preference that was also mentioned by physicians in our study). Another study [18], which concerned aspects for consideration in disability evaluation, suggested that the *timespan* covered in the evaluation (e.g., 5 days, 3 months, or 5 years) determined the value of the aspects used: For longer timespans, less relevance was attributed to patient-related factors. Some physicians in our study made explicit comments that in vocational rehabilitation settings, they would attribute more value to those patient-related prognosis aspects and that these could be targeted by interventions. Prognostic systematic reviews have shown that, regardless of context, patient-related factors, such as coping and self-efficacy, have more prognostic value for participation in work than factors of a medical nature [19–22] and could inform prognosis assessment and re-evaluations over time.

#### The importance of the six areas of prognosis: implicit role of physician-related considerations

Physician-related considerations were regarded by the physicians in our study as often subconscious factors that influence the physician’s judgment. The consequences of those influences for prognosis assessment are mentioned in various medical studies within several fields [8, 23–25]. For example, physicians tend to express the prognosis in a way that is too optimistic. This could originate from, for example, a tendency to provide hope or to stimulate healthy rehabilitation and recovery behavior [8] and “not to harm” by taking those away [8, 24]. For example, an earlier study found that physicians performing disability assessments did not want to permanently deny young adults any hope or chance of future work participation [15], given the positive aspects of work. To overcome some of those consequences, physicians in our study said that the important thing is to be aware of these influences.

#### The way to support physicians: EBM as core ingredient, covering relevant prognosis aspects

The physicians in this study wanted support, including evidence-based prognostic information, preferably pre-appraised. Evidence should ideally be tailor-made, as suggested by earlier studies [7, 8]. Even if prognostic evidence is present, it requires skills to find, appraise, and apply it in a particular case and within the country-specific legislative context [26–28]. However, studies have demonstrated that training in evidence-based medicine may improve the quality of disability evaluations, prognosis assessment, and job satisfaction [4, 29]. Some physicians in our study mentioned that useful prognostic search strings might be provided, thus meeting the demand for help in finding prognostic evidence. This demand was also reported in other studies [26, 30], which led to research providing potential search strategies, filters, or strings regarding themes such as prognosis and work participation [30–32]. The desire for user-friendliness, simplicity, and help in overseeing the various prognostic aspects was also identified by Kox et al. [8] and Louwerse et al. [33], both of whom were exploring possible prognostic tools. In contrast, when presented with possible prognostic tools, physicians also stressed the importance of preserving their professional autonomy to make unique, tailored evaluations. [34, 35] Moreover, they needed to become acquainted with them and they wanted to estimate their validity. [35]

### Strengths and limitations

A strength of our study is that it combined insights from the quantitative data with qualitative data from corresponding remarks from physicians. However, our questionnaire was not suited for an in-depth exploration of the reasons why the importance was scored higher or lower. Also, we cannot exclude a selection effect, as the physicians attending the workshop may be more interested in this topic, but it is not clear how or in which direction this could have influenced the results.

The fact that the case vignette concerned a clear, severe medical condition might explain why the physicians did not elaborate much on the functional abilities of the patient. Some commented that this patient had no abilities for work at all and referred to the medical condition. It would be useful to evaluate the importance of prognostic aspects in a similar study that includes a case vignette with a less severe, chronic health condition (e.g., rheumatoid arthritis) or a condition with less medical clarity (e.g., MUPS). However, we tried to partially counter this disadvantage by asking for opinions on the prognosis for “general” cases, although we acknowledge that what is “general” could mean different things for the participants in this study.

### Conclusions

This study demonstrated that all six areas of prognosis are important and that their individual contribution during prognosis assessment may vary from case to case. There is a need for evidence-based prognostic decision-making as well as tools to assist physicians in searching for, appraising, and applying prognostic evidence to substantiate their prognosis assessments.

## Data Availability

The datasets generated during and/or analyzed during the current study are available from the corresponding author on reasonable request.

## Abbreviations

ADP(s): Aspect(s) of disability prognosis
EBM: Evidence-based medicine
ICF: International Classification of Functioning, Disability and Health
IQR: Interquartile range
MSA(-P): Multiple system atrophy (parkinsonism)
MUPS: Medically unexplained physical symptoms
WMA: World Medical Association
